# Glycogenic Hepatopathy in Type 1 Diabetes Mellitus

**DOI:** 10.1155/2015/236143

**Published:** 2015-08-10

**Authors:** Murat Atmaca, Rifki Ucler, Mehmet Kartal, Ismet Seven, Murat Alay, Irfan Bayram, Sehmus Olmez

**Affiliations:** ^1^Department of Endocrinology and Metabolism, Yuzuncu Yil University Faculty of Medicine, 65100 Van, Turkey; ^2^Department of Internal Medicine, Yuzuncu Yil University Faculty of Medicine, 65100 Van, Turkey; ^3^Department of Pathology, Yuzuncu Yil University Faculty of Medicine, 65100 Van, Turkey; ^4^Department of Gastroenterology, Yuzuncu Yil University Faculty of Medicine, 65100 Van, Turkey

## Abstract

Glycogenic hepatopathy is a rare cause of high transaminase levels in type 1 diabetes mellitus. This condition, characterized by elevated liver enzymes and hepatomegaly, is caused by irreversible and excessive accumulation of glycogen in hepatocytes. This is a case report on a 19-year-old male case, diagnosed with glycogenic hepatopathy. After the diagnosis was documented by liver biopsy, the case was put on glycemic control which led to significant decline in hepatomegaly and liver enzymes. It was emphasized that, in type 1 diabetes mellitus cases, hepatopathy should also be considered in the differential diagnoses of elevated liver enzyme and hepatomegaly.

## 1. Introduction

Liver enzyme elevation is more common among diabetic patients compared to the general population. This condition is often associated with nonalcoholic hepatosteatosis [1, 2]. Another very rare cause of elevated liver enzymes, especially among type 1 diabetic patients, is glycogenic hepatopathy (GH). GH develops due to excessive and irreversible accumulation of glycogen in the hepatocytes and causes liver function disorders and hepatomegaly [3, 4]. Mauriac first defined GH in a child with brittle diabetes, as a component of Mauriac syndrome, characterized by delayed development, hepatomegaly, cushingoid appearance, and delayed puberty [5]. Additionally, GH can also be observed in adult type 1 diabetic individuals without other components of Mauriac syndrome [6–8]. Hyperglycemia and overinsulinization (poor glycemic control) are believed to be metabolic preconditions in GH. GH therapy is performed via establishing glycemic control. Tight glycemic control via intensive insulin therapy provides full remission of clinical, laboratory, and histological abnormalities [4]. Here, a 19-year-old case diagnosed with GH is presented with a discussion referenced to the medical literature.

## 2. Case

A 19-year-old male patient was admitted to the emergency department due to loss of appetite and nausea complaints that continued for two days. The medical history of the case showed that he was followed up due to type 1 diabetes for 8 years and for hepatosteatosis for 3 years, had poor blood glucose regulation despite insulin analogue and basal insulin therapy, and was hospitalized and followed up 8–10 times for diabetic ketoacidosis. He did not have any outstanding condition in family history. His arterial blood pressure was measured as 90/50 mmHg and pulse as 92 beat/min and body temperature was 36.9°C in his physical examination. At his abdominal examination, liver was palpable 4 cm below the costal margin, and no splenomegaly or acid was determined. The cardiovascular and respiratory system examination results were normal. The laboratory results of the case obtained in the emergency department were as follows: glucose 350 mg/dL, aspartate aminotransferase (AST) 603 IU/mL, alanine aminotransferase (ALT) 570 IU/mL, alkaline phosphatase (ALP) 921 U/L, and gamma glutamyl transferase (GGT) 379 U/L. Ketone was positive in his full urinalysis but there was no evidence of acidosis in his arterial blood sample (pH: 7.38; bicarbonate 22 mEq/L). The case was hospitalized with diabetic ketosis and liver function disorder diagnoses and was put on intravenous liquid and insulin replacement for the treatment of diabetic ketosis. On the 36th hour of his hospitalization, urine ketone was negative and nausea had regressed. The patient was then started on intensive insulin therapy. He had elevated aminotransferase and hepatomegaly. Abdominal ultrasonography results yielded the fact that the liver was 24 cm and the parenchyma appeared concordant with grade II hepatosteatosis.

**Figure 1:**
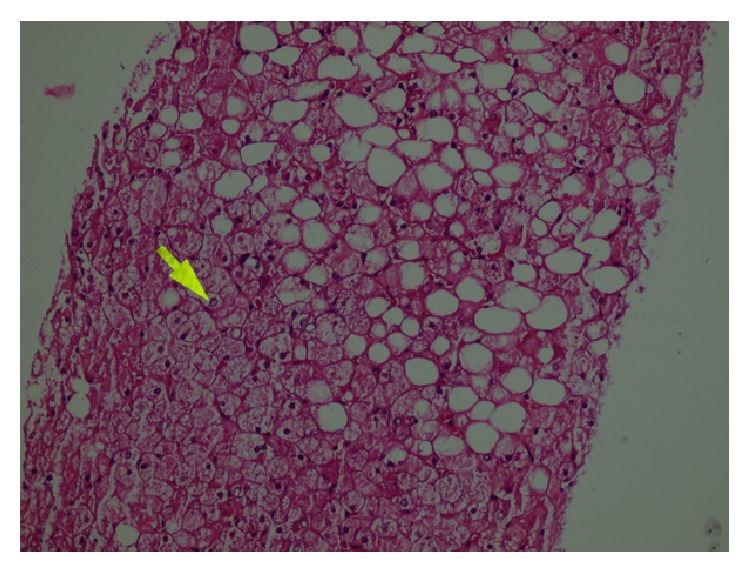
Histological findings in the liver (hematoxylin and eosin stain). The hepatocytes are diffusely swollen with rarefaction of cytoplasm and accentuation of the cell membranes. Numerous hepatocytes exhibit glycogenated nuclei. There are fatty droplets presenting without inflammation and fibrotic changes.

**Figure 2:**
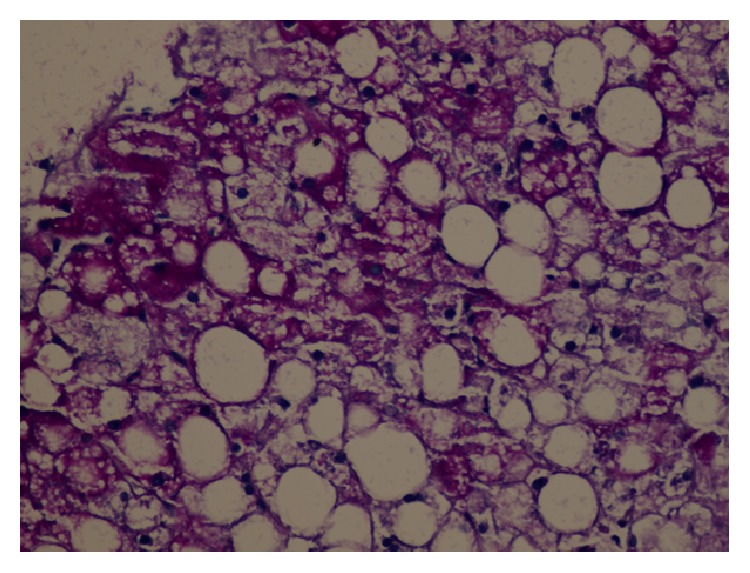
Histological findings in the liver (Periodic Acid-Schiff (PAS) stain). Cross section without showing zonal distribution pattern, having the macrovesicular steatosis and hydropic degeneration. In some areas PAS histochemical analysis showed hepatocyte cytoplasm filled with glycogen and glycogenic core (PAS, original size ×40).

**Figure 3:**
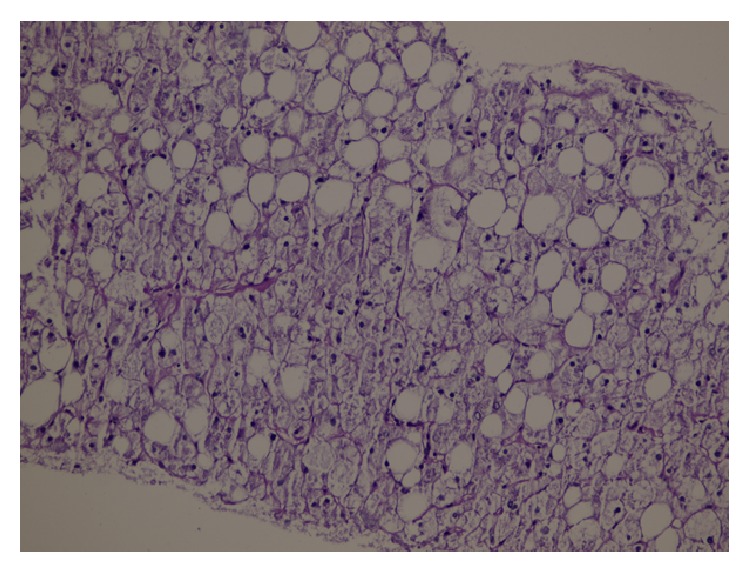
Histological findings in the liver (Periodic Acid-Schiff (PAS) stain) after diastase digestion. The hepatocyte cytoplasm is not stained with PAS.

The history of the case included elevated liver enzymes for 3 years and hepatosteatosis. It was recorded that the liver enzymes had great fluctuations within the past 3 years, he was hospitalized due to hyperglycemia or ketoacidosis, and his liver enzymes declined when his blood sugar was regulated. The patient who did not have a history of hepatotoxic agent use was examined with regard to chronic hepatitis differential diagnosis since the aminotransferase levels were found to be elevated to more than 10 times the normal values, intermittently for 3 years. Serologic and biochemical investigations for chronic viral hepatitis, hemochromatosis, Wilson's disease, autoimmune hepatitis, and/or overlap syndromes had normal results (Table 1). Thereupon, a liver biopsy was performed. Significant widespread macrovesicular fatty changes in the hepatocytes and focal intranuclear clearing were observed in the histopathological examinations. No fibrosis or inflammation was detected (Figure 1). Strong intracytoplasmic PAS staining was observed in favor of glycogen in the histochemical investigations (Figure 2). After diastase digestion, which selectively degrades glycogen, PAS staining was no longer positive, confirming that glycogen accumulation was responsible for the findings (Figure 3).

The case was diagnosed with GH due to the present clinical, laboratory, and pathological findings. A significant decline was observed in the liver enzymes (aspartate aminotransferase (AST) 77 IU/mL, alanine aminotransferase (ALT) 73 IU/mL, alkaline phosphatase (ALP) 139 U/L, and gamma glutamyl transferase (GGT) 133 U/L) and the liver size (measured as 19 cm in the control abdominal ultrasonography) with a 3-week diet and intensive insulin therapy (1 IU/kg).

## 3. Discussion

The prevalence of elevated liver enzymes has increased among diabetic patients. The prevalence of elevated alanine aminotransferase level is 9.5% among type 1 and 12.1% among type 2 diabetics. These percentages are higher than those expected in a general population (2.7%) [9, 10]. Nonalcoholic fatty liver disease (NAFLD) is the most common cause of chronic liver disease for both the general and the diabetic populations today. Obesity and metabolic syndrome play major roles in NAFLD pathogenesis. Therefore, it is observed more frequently in type 2 diabetes cases compared to type 1 [11]. On the other hand, it should be noted that NAFLD is a diagnosis of exclusion [12]. The case presented was followed up for the past three years with NAFLD diagnosis due to elevated liver enzyme levels and ultrasonographic hepatosteatosis appearance. The extreme elevation (over 10 times the normal values) of the liver enzyme levels of our case, who was a type 1 diabetic, was a trigger for investigating secondary causes. Autoimmune hepatitis, which can accompany toxic hepatitis, chronic viral hepatitis, Wilson's disease, hemochromatosis, alpha-1 antitrypsin deficiency, and type 1 diabetes, was ruled out serologically and biochemically. Afterwards, the case was diagnosed with GH upon liver biopsy.

GH, a disease that develops due to hepatic glycogen accumulation, is characterized by hepatomegaly and elevated liver enzyme levels [13–15]. GH was first defined as glycogen accumulation in 1930, as a component of Mauriac syndrome (type 1 diabetes, delayed development, hepatomegaly, cushingoid appearance, and delayed puberty) [5]. However, GH can also be observed in adult type 1 diabetic individuals without other components of Mauriac syndrome [6, 7]. Type 1 diabetes patients compose majority of the case reports on this rare condition. At the same time, there are reports in the medical literature of development of this condition in three children who were not diabetic and in one dumping syndrome case that did not have glucose intolerance [16–19].

Hyperglycemia and overinsulinization are believed to be metabolic preconditions for hepatic glycogen accumulation in GH. Hyperglycemia activates glycogen synthase by inhibiting glycogenesis via glycogen phosphorylation inactivation. Glycogen accumulation further increases because insulin also activates glycogen synthase [20]. A study conducted in rats with insulin deficiency has shown that, after a single dose of insulin injection, glycogenesis continues for a significant amount of time after blood glucose levels return to the preinjection levels [21]. In other words, hepatic glycogen accumulation occurs despite the high cytoplasmic glucose concentration in the presence of insulin. Therefore, frequent hyperglycemic episodes and the following insulin therapies are believed to be the primary pathogenetic mechanisms of hepatomegaly and liver function disorder that develop in type 1 diabetic patient due to glycogen accumulation. Yet, it is not clear why this pathogenetic mechanism develops in a small patient group. One of the hypotheses on this matter was the defect in the genes that code the proteins that regulate the glycogen synthase and/or glucose 6-phosphatase activity [22]. GH therapy is performed via establishing glycemic control. Tight glycemic control, provided via intensive insulin therapy, results in full remission of clinical, laboratory, and histologic abnormalities [4]. It has been reported in the medical literature that remission was attained in a case with GH by a continuous insulin infusion pump implanted under the skin [7]. Similarly, the case presented here also attained blood glucose regulation, accompanied by reduction in the liver size and significant decreases in ALT and AST levels with intensive insulin therapy. Control liver biopsy was not performed in this case after laboratory and radiological improvements; therefore, histologic remission was not valuated. The fact that the liver enzymes could not return to the normal ranges was believed to be associated with the hepatosteatosis accompanying GH in this case.

Consequently, liver enzyme elevation is a common condition among diabetic patients. Though often NAFLD is responsible for this condition, in case of type 1 diabetic patients, GH should be considered for differential diagnosis in the presence of severe elevation of aminotransferase levels.

## Figures and Tables

**Table 1:** Laboratory tests conducted for differential diagnosis of chronic hepatitis.

Parameter	Results
HbSAg	Negative
Anti-Hbc IgG	Negative
Anti-HCV	Negative
Ferritin	200 ng/mL
Iron	41 *µ*g/dL
Total iron binding capacity	216 *µ*g/dL
Urine Cu/24 hours	17 *µ*g
Ceruloplasmin	35 mg/dL
Alpha-1 antitrypsin	123 mg/dL
ANA (anti-nuclear antibody)	Negative
AMA M2 (anti-mitochondrial antibody)	Negative
Anti-SMA (anti-smooth muscle antibody)	Negative
ANCA (anti-nuclear cytoplasmic antibody)	Negative
